# Not All Young Journals Are Predatory

**DOI:** 10.5811/westjem.2016.10.32826

**Published:** 2016-12-06

**Authors:** Adam Singer

**Affiliations:** Stony Brook University, Department of Emergency Medicine and Editor, Clinical and Experimental Emergency Medicine, Stony Brook, New York

I read the article by Hansoti et al. with great interest in which they list “predatory” open access emergency medicine journals.^1^ Unfortunately, the authors neglected to mention a major limitation of their study methodology. The process required for a new journal to be included in various recognized medical library indexing services such as PubMed or the Scientific Citation Index is often complex and lengthy, sometimes requiring several years before being included. Thus, lack of inclusion of a journal title within these search engines is not evidence that the journal is illegitimate, since it may be too young to be included. Therefore, I was disappointed to see the journal *Clinical and Experimental Emergency Medicine* among the list of so-called “predator” journals. *Clinical and Experimental Emergency Medicine* (*CEEM*) is non-for-profit, peer reviewed and the official English language journal of the Korean Society of Emergency Medicine inaugurated about two year ago. The journal does not charge publication fees and is funded by the Korean Society. The journal has just been included into PubMed. The Korean Society of Emergency Medicine represents hundreds of Korean emergency physicians and is a highly reputable organization. Korean emergency physicians have made significant contributions to the body of emergency medicine and acute care knowledge, some of which have been published in *CEEM* as well as many other well-established journals. The editorial board of *CEEM* includes multiple internationally renowned emergency physicians who have joined forces to support the efforts of the Korean Society. *CEEM* was established as a platform for a large number of Asian emergency physicians to highlight many of the issues unique to this region. In today’s era of emergency medicine globalization and rapid international growth it is important for all of us to come together and support the efforts of national emergency medicine organizations to grow their clinical and academic missions, such as the establishment of new journals like *CEEM*. Thus, extreme care should be taken before prematurely labeling young yet perfectly legitimate journals as “predators,” especially in our relatively young field of emergency medicine.

## Figures and Tables

**Table 4:** Open access emergency medicine journals that have achieved indexing in recognized services and are therefore legitimate rather than predatory. NLM (National Library of Medicine) Catalog, SJR (Scimago Journal Rank), DOAJ (Director of Open Access Journals), EBSCOhost journal master list, and WS (Thomson Reuters Web of Science, including “expanded” and “emerging sources”).

Journal title and weblink		NLM catalog	PubMed central	SJR	DOAJ	EBSCOhost	WS
1.	Advances in Emergency Medicine from Hindawi http://www.hindawi.com/journals/aem/contents/				x		
2.	African Journal of Emergency Medicine^a^ http://www.afjem.org/	x		x	x		x
3.	Australian Journal of Emergency Management^b^ https://ajem.infoservices.com.au/items/AJEM-31-02			x			x
4.	BMC Emergency Medicine https://bmcemergmed.biomedcentral.com/	x	x	x	x	x	x
5.	Bulletin of Emergency & Trauma http://www.beat-journal.com/BEATJournal/index.php/BEAT	x	x		x		
6.	Case Reports in Emergency Medicine from Hindawi Publishing Corporation^c^ http://www.hindawi.com/journals/	x	x		x	x	
7.	Clinical and Experimental Emergency Medicine^d^ http://www.ceemjournal.org/	x	x				
8.	EAJEM: Eurasian Journal of Emergency Medicine http://www.akademikaciltip.com/eng/Anasayfa	x	x				x
19.	Emergency: An Academic Emergency Medicine Journal http://journals.sbmu.ac.ir/emergency	x	x		x		
10.	Emergency Care Journal http://www.pagepressjournals.org/index.php/ecj				x		x
11.	Emergency Medicine International from Hindawi Publishing Corporation http://www.hindawi.com/journals/emi/	x	x		x	x	x
12.	Hong Kong Journal of Emergency Medicine http://www.hkjem.com/			x			x
13.	International Journal of Emergency Medicine from Springer Open https://intjem.springeropen.com/	x	x	x	x	x	x
14.	Iranian Journal of Emergency Medicine http://www.journals.sbmu.ac.ir/en-iranjem				x		
15.	ISRN Emergency Medicine http://www.hindawi.com/jour-nals/isrn/contents/emergency.medicine/				x	x	
16.	Journal of Cardiovascular Emergencies http://www.degruyter.com/view/j/jce				x		
17.	Journal of Emergencies, Trauma, and Shock http://www.onlinejets.org/	x	x	x	x	x	
18.	Journal of Emergency Medicine, Trauma and Acute Care from Qscience.com http://www.qscience.com/loi/jemtac			x	x		
19.	Journal of Emergency Practice and Trauma http://jept.ir/				x		
20.	Journal of Trauma Management and Outcome^e^ https://traumamanagement.biomedcentral.com/	x	x	x	x	x	
21.	Open Access Emergency Medicine from Dovepress^f^ https://www.dovepress.com/open-access-emergency-medicine-journal	x	x	x	x		x
22.	Scandinavian Journal of Trauma, Resuscitation and Emergency Medicine^g^ http://sjtrem.biomedcentral.com/	x	x	x	x		x
23.	Turkish Journal of Emergency Medicine http://www.trjemergmed.com/	x	x				x
24.	Western Journal of Emergency Medicine: Integrating Emergency Care with Population Health http://escholarship.org/uc/uciem_westjem	x	x	x	x	x	x
25.	World Journal of Emergency Medicine^h^ http://www.wjem.org/	x	x			x	x
26.	World Journal of Emergency Surgery^i^ http://wjes.biomedcentral.com/	x	x	x	x	x	x

Journal numbers highlighted in yellow are changed from the original published version.

a African Journal of Emergency Medicine was recently added to NLM Catalog and only selected citations will soon be in PubMed. The full-text content currently is not in PMC.

b Australian Journal of Emergency Management was incorrectly placed in the potential predatory journal list. The journal is found and/or indexed in SJR and WS.

c Case Reports in Emergency Medicine from Hindawi Publishing Corporation is also found in the EBSCOhost journal master list.

d Clinical and Experimental Emergency Medicine has recently been added to the NLM Catalog. The full-text content is now archived in PMC and the citations are now searchable in PubMed.

e Journal of Trauma Management and Outcomes is found and or indexed in all the selected databases except WS.

f Open Access Emergency Medicine from Dovepress was incorrectly marked in EBSCOhost.

g Scandinavian Journal of Trauma, Resuscitation and Emergency Medicine is indexed in WS, but the search for Thomson Reuters indexed journals needs to include “&” symbol rather than “and” in the title, in order to find the journal’s title.

h Both the World Journal of Emergency Medicine and the World Journal of Emergency Surgery are found and/or indexed in the EBSCOhost and WS.

**Table 5:** Emergency medicine journals that have not achieved indexing in any recognized service, and are therefore potential or probable predatory open-access journals.

Journal title and weblink	
1.	Archives of Emergency Medicine and Critical Care from SciMed Central i http://www.jscimedcentral.com/EmergencyMedicine/
2.	Austin Emergency Medicine http://austinpublishinggroup.com/emergency-medicine/
3.	Edorium Journal of Emergency Medicine http://www.edoriumjournalofemergencymedicine.com/about-us/about-us.php
4.	Emergency Medicine: Open Access^j^ http://www.omicsgroup.org/journals/emergency-medicine.php
5.	Emergency Medicine Open Journal from Openventio Publishers http://openventio.org/OpenJournal/EmergencyMedicine.html http://openventio.org/index.php
6.	Gavin Journal of Emergency Medicine^k^ (journal has not published any issues) http://gavinpublishers.org/index.php/emergency-medicine
7.	Henry Journal of Emergency Medicine, Trauma & Surgical Care (journal has not published any issues) http://www.henrypublishinggroup.com/index.php/emergencymedicine/about
8.	HSOA Journal of Emergency Medicine, Trauma & Surgical Care http://www.heraldopenaccess.us/journals/Emergency-Medicine-Trauma-&-Surgical-Care/
9.	International Journal of Emergency Mental Health and Human Resilience^l^ http://www.omicsonline.com/open-access/international-journal-of-emergency-mental-health-and-human-resilience.php
10.	Internet Journal of Emergency Medicine http://ispub.com/IJEM from Internet Scientific Publications.
11.	Journal of Emergency Medicine and Intensive Care http://elynsgroup.com/journal/journal-of-emergency-medicine-and-intensive-care
12.	Journal of General and Emergency Medicine http://scientonline.org/journals/general-emergency-medicine/31
13.	Journal of Intensive Care and Emergency Medicine http://www.signavitae.com/
14.	Mathews Journal of Emergency Medicine http://www.mathewsopenaccess.com/EMedicine.html
15.	OA Emergency Medicine http://www.oapublishinglondon.com/oa-emergency-medicine
16.	Open Emergency Medicine (latest content is 2013) http://benthamopen.com/toemj/home
17.	Pediatric Emergency Care and Medicine: Open Access http://pediatric-emergency-care.imedpub.com/
18.	SM Emergency Medicine and Critical Care (SMEM) http://smjournals.com/emergency-medicine/
19.	The Scientific Pages of Emergency Medicine http://thescientificpages.org/page/general-medicine/scientific-pages-of-emergency-medicine.php
20.	Trauma and Emergency Care (TEC) from OAT (Open Access Text) http://oatext.com/Trauma-and-Emergency-Care-TEC.php

Journal numbers highlighted in yellow are changed from the original published version.

i *Archives of Emergency Medicine and Critical Care* has been listed recently in the NLM Catalog, but “only citations for author manuscripts are included”. It appears an author deposited the article to PubMed Central. Thus only this citation is found in PubMed under this journal. *JSciMed Central* published this journal and apparently is a well-known predatory publisher (https://scholarlyoa.com/2014/06/24/real-location-of-jscimed-central-revealed/).

j *Emergency Medicine: Open Access* – This journal has not achieved indexing in any recognized service. A record is found in the NLM Catalog where it said “Only citations for author manuscripts are included.” It appears one author deposited an article to PubMed Central. This is the only citation found in PubMed under this journal. After reviewing the publisher’s website, we determined this is most likely a predatory journal.

k *Gavin Journal of Emergency Medicine* (journal has not published any issues) http://gavinpublishers.org/index.php/emergency-medicine - This journal is now published under a new title, Emergency Medicine Investigations, with an new URL under.com rather than.org.

l *International Journal of Emergency Mental Health and Human Resilience* was incorrectly marked indexed in WS. The journal was indexed in MEDLINE from Winter 1999 to Winter 2014. Only selected citations after Winter 2014 are in PubMed. In 2013 the journal was sold to OMICS Publishing Group, which was recently sued by the U.S. Federal Trade Commission for deceptive practices in August 2016.

**Table 6:** Emergency medicine journals that appear legitimate, but have not achieved indexing in any recognized service.

Journal title and weblink	
1.	Emergency Medicine and Health Care from HOAJ (Herbert Open Access Journal) http://www.hoajonline.com/emergmedhealthcare http://www.hoajonline.com/)
2.	Frontiers in Public Health | Disaster and Emergency Medicine^m^ http://journal.frontiersin.org/journal/public-health/section/disaster-and-emergency-medicine
3.	International Journal of Critical Care and Emergency Medicine http://clinmedjournals.org/International-Journal-of-Critical-Care-and-Emergency-Medicine.php
4.	Journal of Japanese Society for Emergency Medicine https://www.jstage.jst.go.jp/browse/jsem
5.	Journal of Emergency Medicine & Critical Care from Avens Publishing Group Inviting Innovations http://www.avensonline.org/medical/emergency-medicine-and-critical-care/home-5/
6.	Open Journal of Emergency Medicine from Scientific Research An Academic Publisher http://www.scirp.org/journal/ojem/

Journal numbers highlighted in yellow are changed from the original published version.

m Frontiers in Public Health | Disaster and Emergency Medicine appears to be a new journal subsidiary to Frontiers in Public Health (http://journal.frontiersin.org/journal/public-health), which is found in DOAJ, but the new journal, “Frontiers in Public Health | Disaster and Emergency Medicine” is not. The core journal has 858 articles published, while the new journal only has 19 at press time. This new journal is not now found in any indexing services.
