# Supplementary material for: Not All Young Journals Are Predatory
Source: West J Emerg Med. 2016 Dec 6;18(2):318. doi: 10.5811/westjem.2016.10.32826 (PMC5321261; doi:10.5811/westjem.2016.10.32826)
Supplement: Related texts

# In reply to: “Not All Young Journals Are Predatory”

**Published:** 2016-12-06

**Authors:** Linda S. Murphy, Bhakti Hansoti, Mark I. Langdorf

**Affiliations:** * University of California, Irvine, Libraries, Irvine, California; † Johns Hopkins University, Department of Emergency Medicine, Baltimore, Maryland; ‡ University of California, Irvine, Department of Emergency Medicine, Irvine, California

Dear Dr. Singer,

We greatly appreciate your interest and comment on our study.^1^ Your point about lack of inclusion of a young open access (OA) journal is well taken. We can’t agree with you more that the process of getting a new journal recognized and included in various indexing services is both lengthy and complex. It took *WestJEM* five years and three attempts with major improvement prior to acceptance to MEDLINE.^2^ We regret that we neglected to include this important issue as one of the limitations in our study methodology.

While the methodology we chose to use in our study was imperfect, we did address several limitations. Our intention was to present the likely predatory vs. legitimate OA journal titles and their website links as aids to authors to further scrutinize the journal before submission. As indicated, *Clinical and Experimental Emergency Medicine (CEEM)* was not found in any of the selected directories, indexes, databases, and publishers that we searched at the time the study was conducted. We are happy to discover that *CEEM* is now listed in the National Library of Medicine (NLM) Catalog. The detail record (https://www.ncbi.nlm.nih.gov/nlmcatalog/?term=clinical+experimental+emergency+medicine) shows *CEEM* content is now archived and accessible in PubMed Central (PMC) (https://www.ncbi.nlm.nih.gov/pmc/journals/3081/). As a result, its abstracts are automatically migrated to PubMed as well. This is an important milestone for *CEEM* to be recognized by NLM and we congratulate the Editorial Board for a major accomplishment.

We also did a thorough review of the current *CEEM* website (http://ceemjournal.org/). It is well designed, clear, and meets all the criteria for a legitimate, official journal website. Its information includes the 12 questions that our study posted for readers and researchers to be considered when reviewing a journal or publisher’s website. Interestingly, *CEEM* is a unique OA journal that does not charge authors any processing fee. *“There is no author’s submission fee or other publication-related fee since all costs of the publication process are underwritten by the Korean Society of Emergency Medicine* (http://ceemjournal.org/authors/authors.php).”

Your letter and another inquiry led us to check and update the information included in the original tables 4 and 5 in the manuscript. We found some changes, now reflected in the updates below (accessed November 25, 2016). Changed information in the tables are highlighted in yellow, and we have added explanatory footnotes. We also created a Table 6 (not in the original article) which lists journals that appear legitimate, but have not achieved indexing in any recognized service.

Again, thank you for bringing this oversight to our attention. In the spirit of open access, we encourage other legitimate scientists and publishers to expand the space for emergency medicine scholarship, so research and best practice can be freely available to the developed and developing world.

Sincerely,

Linda S. Murphy, MLIS, Research Librarian for the Health Sciences, University of California, Irvine Libraries
Bhakti Hansoti, MBChB, MPH, Johns Hopkins University, Department of Emergency Medicine, Baltimore, Maryland
Mark I. Langdorf, MD, MHPE, University of California, Irvine, Department of Emergency Medicine, Irvine, California

## References

[1] Hansoti B, Langdorf MI, Murphy LS (2016). Discriminating Between Legitimate and Predatory Open Access Journals: Report from the International Federation for Emergency Medicine Research Committee. West J Emerg Med.

[2] Forney K, Murphy LS (2016). Getting found: Indexing and the Independent Open Access Journal. West J Emerg Med.
